# "Do I really want to do this?" Longitudinal cohort study participants' perspectives on postal survey design: a qualitative study

**DOI:** 10.1186/1471-2288-11-8

**Published:** 2011-01-27

**Authors:** Helen Harcombe, Sarah Derrett, Peter Herbison, David McBride

**Affiliations:** 1 Department of Preventive and Social Medicine, University of Otago, New Zealand; 2 Injury Prevention Research Unit, Department of Preventive and Social Medicine, University of Otago, New Zealand

## Abstract

**Background:**

Randomised controlled trials have investigated aspects of postal survey design yet cannot elaborate on reasons behind participants' decision making and survey behaviour. This paper reports participants' perspectives of the design of, and participation in, a longitudinal postal cohort survey. It describes strengths and weaknesses in study design from the perspectives of study participants and aims to contribute to the: 1) design of future cohort surveys and questionnaires generally and, 2) design of cohort surveys for people with musculoskeletal disorders (MSDs) specifically.

**Methods:**

In-depth interviews explored the design of postal surveys previously completed by participants. Interviews used open ended questioning with a topic guide for prompts if areas of interest were not covered spontaneously. Thematic data analysis was undertaken based on the framework method. A second researcher verified all coding.

**Results:**

Data from fourteen interviews were analysed within three main themes; participation, survey design and survey content. One of the main findings was the importance of clear communication aimed at the correct audience both when inviting potential participants to take part and within the survey itself. Providing enough information about the study, having a topic of interest and an explanation of likely benefits of the study were important when inviting people to participate. The neutrality of the survey and origination from a reputable source were both important; as was an explanation about why information was being collected within the survey itself. Study findings included participants' impressions when invited to take part, why they participated, the acceptability of follow-up of non-responders and why participants completed the follow-up postal survey. Also discussed were participants' first impression of the survey, its length, presentation and participants' views about specific questions within the survey.

**Conclusions:**

Ideas generated in this study provide an insight into participants' decision making and survey behaviour and may enhance the acceptability of future surveys to potential participants. As well as clear communication, participants valued incentives and survey questions that were relevant to them. However, opinions varied as to the preferred format for responses with some advising more opportunity for open-ended feedback. We also found that some standard format questions can raise quandaries for individual participants.

## Background

Health researchers, undertaking research amenable to quantitative analysis, once tended to emphasise data collected from clinical records (e.g. numbers of treatments or hospital readmissions) or from observations by health professionals (e.g. participants' abilities performing functional tasks measured on clinician-administered tools). In the latter part of the last century, researchers became concerned that morbidity, mortality and hospital admissions and discharges data, did not always provide sufficient information to determine incidence, prevalence and outcome [1]. Researchers began to include measures which collected data directly from participants (e.g. measures of self-reported function and health outcome). In the 1980s caution was urged to ensure that such self-reported measures were meaningful, valid and reliable [2].

When collecting self-reported data from participants, researchers use a variety of methods including: telephone interviews, in-person interviews, web-based questionnaires and postal paper-based questionnaires. Different modes of data collection, with consequent effects on response proportions, also raise issues about potential bias, particularly in studies assessing exposure to potential risk factors [3-5].

A recent systematic review identified 481 randomised controlled trials (RCTs) which had evaluated 110 different methods aimed at increasing the response rate to postal questionnaires [6,7]. Factors found to increase participation include: monetary incentives, using potential participants' names when making contact, handwritten researcher signatures on the introductory letter, pre-questionnaire contact, placing more relevant and easier-to-answer questions near the beginning of the questionnaire and having the initial approach coming from a university rather than from a governmental or commercial organisation.

Despite the number of trials already undertaken, further evidence is called for "...that might increase the quality and quantity of the data collected by questionnaire, and of participation in trials more generally" (p. 7) [6]. Reasons underlying participants' decision-making and survey behaviours cannot be sufficiently elaborated in RCTs. Criticism has also been levelled at the lack of theoretical frameworks for understanding study recruitment methods and questionnaire design [8,9].

Qualitative research offers opportunities to explore with participants the reasons behind survey behaviour. To date, few studies appear to have investigated, in a qualitative manner, the perspectives of participants in postal surveys; although Nakash et al (2008) have reported results of a nested qualitative study which explored reasons for participation, and non-participation, in a clinical trial of mechanical supports for severe ankle sprain [10].

This paper reports study participants' views about the design of, and participation in, a longitudinal postal cohort survey investigating the prevalence and incidence of musculoskeletal disorders (MSDs). The purpose of the research described in this paper is to increase our understanding of the reasons underlying decisions about participation in longitudinal cohort studies. This may help extend theories about participating in longitudinal cohort surveys, and may also contribute practically with the development of new study questions for future RCTs examining study design. This paper describes strengths and weaknesses in study design from the perspectives of study participants and aims to contribute to the: 1) design of future cohort surveys and questionnaires generally, and 2) design of cohort surveys for people with MSDs specifically.

## Methods

A longitudinal cohort study was undertaken in 2007 and 2008 to investigate musculoskeletal disorders (MSDs) in New Zealand nurses, postal workers and office workers (n = 443). A postal questionnaire was sent to potential participants and, one year later, a follow-up questionnaire was sent to determine incident MSDs and outcomes one year later. The New Zealand cohort study was undertaken as part of an international study of Cultural and Psychosocial Influences on Disability (CUPID). Most questions within the questionnaires were included as part of the CUPID study; some New Zealand specific additions were also included. The baseline survey was a 36-page A4-size booklet and the follow-up survey was a 28-page booklet. Baseline findings from the New Zealand study have been reported previously [11,12]. The main study was approved by the New Zealand Multi-Region Ethics Committee (MEC/06/09/096); the qualitative study reported here was approved by the New Zealand Lower South Regional Ethics Committee (LRS/08/06/021).

In brief, the longitudinal cohort study involved the random selection of 911 nurses, office workers and postal workers from throughout New Zealand. Potential participants were invited to participate via a letter sent to their postal address. This had a time-bound opt out option. Two weeks later, those who had not opted out were sent the baseline postal survey. "Non-responders were sent another copy of the questionnaire after two weeks and were given a telephone reminder at four weeks. Those who were not contactable by telephone were sent a third copy of the questionnaire" (p. 438) [12]. The participation rate of eligible participants was 58% (n = 443). One year later, participants were sent a follow-up postal survey. Follow-up of non-responders took the same format as for the baseline survey. The follow-up rate was 87% (n = 384).

**Figure 1:**
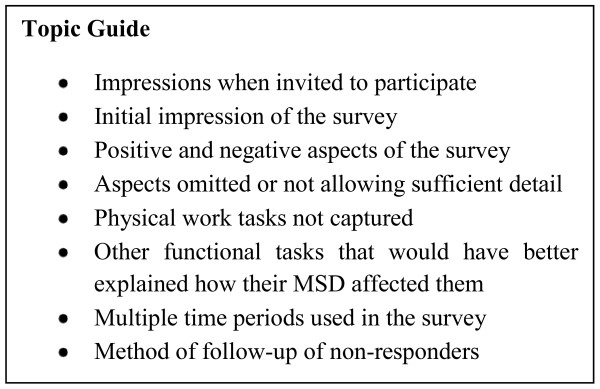
**Topic Guide**.

For the qualitative study reported here, a pragmatic sample consisting of all participants living in the Otago and Southland regions of New Zealand who had reported an MSD in the follow-up postal survey were invited to participate in a single face-to-face qualitative interview in 2008. Interviews were held at a place that was mutually acceptable to participant and interviewer and were digitally recorded for subsequent verbatim transcription. After obtaining consent, participants' were asked a 'prompt' open ended question about their views of the questionnaires, for example "we are really interested in getting feedback about the survey to help [researchers if they] use it [or similar surveys] again in the future, so anything you can tell us about it would be helpful." Participants were given copies of the questionnaires to look through while responding which elicited spontaneous comments. Interviews used open-ended questioning, and participants were free to lead the interview in directions of importance to them. However, certain topic areas were listed on an interview guide and prompts were used if certain topics were not spontaneously covered by participants (Figure 1). For example, not all participants spontaneously commented on the method of contact for the postal survey, or the strategies adopted for follow-up of non-return of the questionnaire; these non-spontaneous responders were then asked open-ended questions about these aspects of the study.

Interview transcripts were analysed using a thematic approach based on the framework method [Ritchie and Spencer, 1994 cited in 13]. NVivo software (version 8) was used to organise the data [14]. Potential themes for analysis were noted during the interviews and when verifying the transcripts. Along with the interview guide, these provided a preliminary thematic framework. The codes in this thematic framework were applied to the interviews one by one. Each piece of text was analysed and grouped into the relevant code. New codes were added, as required, and interviews previously coded were then checked to see if the new code applied. The coding of each interview was checked by a second researcher, with additions made if required. Twenty-eight codes were developed. Following the coding of all interviews these codes were categorised into thirteen areas and then analysed as three main themes. Each piece of data was summarised and the content of each theme considered when developing explanations and implications of the data.

To ensure validity, each stage of the study was discussed closely with a second researcher and two researchers were present at the initial interview. The wider research team comes from a range of clinical and/or research backgrounds. In the interviews, the open question at the start of the interview and having time to browse through the postal surveys meant that participants could discuss aspects that they felt were important. All coding was verified by a second researcher and alternate views were continually sought in the data.

## Results and discussion

Twenty-two people were invited to participate in the qualitative study; fourteen participated, eight declined. Participants were predominantly female (one was male). Participants included all three occupational groups, reported a range of anatomical sites of MSD and had a range of response times to the follow-up postal survey; five responded after the first mail-out, seven after the second mail-out and two after being contacted by telephone (Table 1).

**Table 1:** Interview participants compared to those who were invited but declined

	Participants (n = 14)	Declined (n = 8)
	n	n
**Survey response**		
Early	5	5
Mid	7	2
Late	2	1
**Main MSD**		
Upper limb	9	1
Lower limb	2	2
Neck	2	0
LBP	1	3
Unspecified	0	2
**Occupation**		
Nurse	6	4
Postal worker	3	2
Office worker	5	2
**Sex**		
Male	1	0
Female	13	8
**Age **(years)		
Mean (sd)	45 (7)	41 (13)

**Figure 2:**
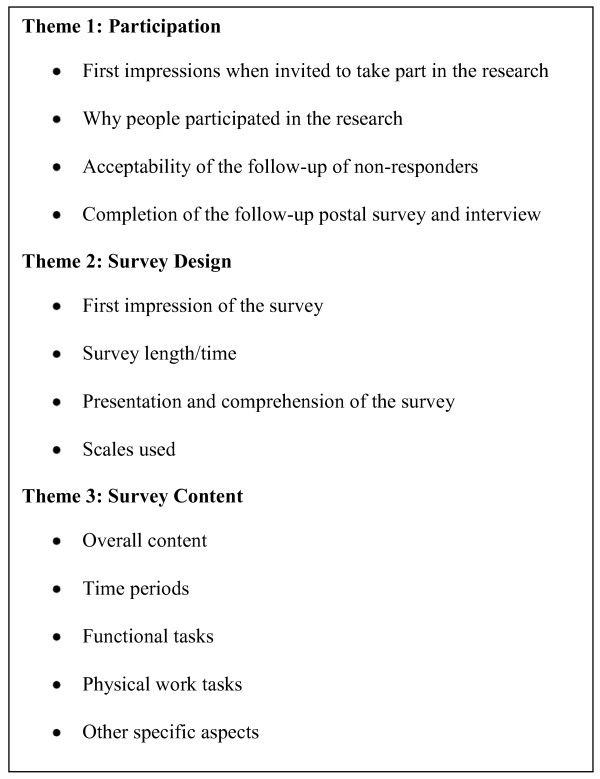
**Themes**.

The three overarching themes identified from analysis of transcripts were participation, survey design and survey content; within each theme are coded categories directly linked to the theme (Figure 2). Findings from the fourteen interviews are presented and discussed under each of the three main themes.

### Theme 1: Participation

We do not know reasons for non-participation among those who declined the invitation to take part in the postal survey. However, this qualitative study provided the opportunity to explore reasons behind the decisions of those who did participate.

#### First impressions when invited to take part in the research

Participants were invited to take part in the research through an information sheet sent to their postal address. Although some first impressions were negative; "Do I really want to do this?" (P6), others were interested in the research as they had experienced an MSD or it aligned with their work or general interest. Several participants took a matter-of-fact approach to completing the survey and "Just filled them out" (P1). However, not all participants were clear how they had been selected for the survey or why their occupational group was being researched. From the perspective of the researchers the selection of participants had been explained in the information sheet, for example for office workers it stated:

'How have my details been obtained?

We have obtained your contact details from the 2005 electoral roll...'

On reflection, however, this states how the person's details have been obtained but not why that person was selected. This was unclear to participants and they made incorrect assumptions that it was through their workplace or treatment providers. There was also a concern about confidentiality despite acknowledging the assurances on the information sheet were adequate; "I felt a little bit exposed, yes I did feel a little bit exposed" (P6). The opportunity to find out more about the study would have been appreciated. For example, an email address or website was a suggestion made by a participant to provide further information to those who wanted it. The terminology 'musculoskeletal disorders' was not clear to all participants. This made us, as researchers, note that the title of the survey on the information sheet and reply-paid envelope used this terminology and, although the first sentence of the information sheet stated "...conditions such as back, neck, upper limb and knee pain" it did not actually specify that these can be known as 'musculoskeletal disorders.' These points emphasise the importance of clear information aimed at the correct audience.

#### Why people participated in the study

A feeling of altruism contributed to participation in the study. Sometimes this was linked to participants' own experience of MSDs; "If this survey is going to help other people stopping it getting to that extent, it would be good" (P5). Among participants there was a sense that MSDs were worthy of study and of interest; "This is actually really quite interesting research" (P10). Altruism, as well as a worthy and pertinent topic, was also important to participants in a survey related to a clinical trial [10]. Some liked the feeling of contributing while others felt a sense of duty; "Oh no, I should" (P13). The influence of their occupational background was apparent for several nurses, for example they had been exposed to colleagues involved in research or the study aligned with their own interests. Other people had time available or enjoyed taking part in surveys. Some felt the study was applicable to them because they had an MSD; "I only filled it in because it was applicable" (P1). However, the survey was intended to be completed regardless of MSD status and if respondents were more likely to have an MSD than non-respondents, the prevalence at baseline could be over-estimated [12].

University sponsorship has previously been found to increase response rates [7]. Here too, where the survey originated was important to people; not all would have participated if the study had come from an organisation they were not happy with. Other reasons contributing to participation were that the survey seemed easy to complete, did not appear to involve much writing or be too long. Both monetary and, to a lesser extent, non-monetary incentives increase response rates [7]. In this study, a tea-bag was included with the questionnaire and, on return of the completed questionnaire, participants were sent a thank-you letter and $10 MTA voucher. Participants liked the inclusion of these incentives and they made it more appealing for people to participate; "The petrol voucher and the tea bag it was, that was incentive enough for me anyway" (P8). The size of the survey meant it required an A4 envelope and a reply-paid envelope was included with the questionnaire. Despite randomised controlled trials showing that reply-paid envelopes do not increase response rates [7], their inclusion in this study was reported to make it easier for participants to return the survey; "It was very easy because you included the envelope..." (P8)

#### Acceptability of the follow-up of non-responders

Follow-up of non responders increases survey response rates [7,15]. In this study, a pre-notification letter (the information sheet) was followed by the questionnaire. Another copy of the questionnaire was sent to non-responders after two weeks and they were telephoned after a further two weeks if a questionnaire had not been received. Those with no telephone number available were sent a third copy of the questionnaire [12]. It is difficult to interpret how acceptable the methods of follow-up were because non-responders were not interviewed. However, for interview participants, these methods were generally acceptable although not everyone would have appreciated a telephone call; "If I hadn't filled it out by the second time... [I was] not going to do it" (P4). However, 41 participants in the follow-up postal survey did return their surveys after being telephoned increasing the follow-up rate by 9%. There was a sense that surveys would be completed straight away or they would be forgotten; "They sit there until you're reminded about them" (P1). Although no statistically significant difference in response between follow-up of non-responders in less than 31 days or between 31 and 60 days has been reported [7], in this study allowing two weeks for returning the surveys was thought to be a good timeframe; "It allowed you time, but not too much" (P10) and "If you haven't done something within two weeks well that's probably gone out of your mind" (P2).

#### Completion of the follow-up postal survey and interview

Some participants had forgotten about the baseline survey when the follow-up questionnaire arrived twelve months later. This could reduce the likelihood of observation bias which can occur when exposures and outcome are measured without blinding (in this case by participant self-report), for example if the participant was more likely to report a certain outcome depending on exposures reported. However, it is unlikely that participants would have an agenda for the survey, making this form of bias improbable. Participants completed the follow-up survey because they felt a sense of duty and could see why they should follow through having entered the study. They felt the baseline survey was a good questionnaire and there was a sense that 'someone' needs to take part in surveys. The interview was not indicated on the initial information about the study as it was proposed later. This did not pose a problem for these participants although we do not have information about those who declined to take part.

### Theme 2: Survey design

#### First impression of the survey

The overwhelming first impression was that the survey was long and this led to concerns; "How much resources are going to have to go into this" (P9) and "Do I really want to do this?" (P10). However many said that, despite their initial impression, once they looked more closely the length was reasonable, particularly as some sections could be missed if they were not applicable. Potential participants who did not look closely at the survey may have decided not to take part on the basis of its lengthy appearance. This was confirmed by one participant who said "I don't know whether some people see it and not open it and go no" (P4). At the outset some were concerned about the time commitment the survey would involve; "I don't think I would have bothered if it looked as though it was going to take me too long" (P8). Those who returned the survey early seemed to find the survey length more acceptable than a participant who returned it after being sent a second survey and having a telephone reminder. Other first impressions were that the survey was surprisingly comprehensive but seemed repetitive in parts as it asked the same questions for different anatomical sites. Some first impressions of the survey were fairly neutral or positive in a non-specific way, others commented that it looked easy to do and was well-presented. Survey length and presentation are discussed in more detail in subsequent sections.

#### Survey length/time

Studies using shorter questionnaires may be more likely to have higher response rates than studies with longer questionnaires [7]. As mentioned, an overwhelming first impression was that it was a long survey although most found it satisfactory to complete once they looked more closely at it.

The time completing the surveys ranged from ten minutes to one hour. Several thought the time it took to do the survey was too long but others thought it was acceptable. That non-applicable sections were able to be missed was important to participants, which made us reflect that letting people know this in the cover letter would convey that the survey was not as long as it first appeared. In the interviews the idea was raised that, in general, surveys can feel longer if the same idea is asked in multiple ways. Previous research also suggests "... that clarity and ease of administration may compensate for questionnaire length" (p. 408) [16]. The MTA voucher and teabag meant the length of the survey was less of a problem for participants; "That was incentive enough for me anyway so I don't think the length of time was really an issue" (P8).

#### Presentation and comprehension of the survey

Participants noted the survey was well-presented and "Clearly set out" (P6) with understandable language and writing that was large enough to read. They liked the inclusion of anatomical site diagrams; "I think the diagrams there make it very clear, they're easy to recognise" (P14) and liked that questions were grouped into sections. Some participants felt their past experience of other surveys meant they had no problems with the current survey. Others commented that questions were "Self-explanatory" (P5), it was "Clear in the instructions" (P7) and "Not difficult" (P13). However for some it was confusing to fill out in parts, for example where the same question was repeated for different anatomical sites of MSDs some felt they had already answered the question but then it was asked for a different body site; "Some of the questions were a little bit difficult to fill out because there seemed to be a bit of repetition in them for me... I think it was by the time I got to the end of the book, it was like, oh now we're in a different part, but there's similar questions" (P10). The idea was raised that the volume of questions and boxes meant the survey was not appropriate for participants who were cognitively impaired. Although some surveys are done online, the written format was preferred; "The ones I do at work are on a computer so I'm kind of staring like this... I could actually sit down with a coffee and do this in my own time" (P11).

#### Scales used

Most of the survey involved ticking or circling an option from a predetermined scale. Opinions about this design varied; if there had been more open ended questions some "Probably wouldn't have returned them" (P4). This aligns with previous findings [7]. However, others would have preferred more open-ended questions; "They're all boxes which is a bit of a nuisance" (P1). The variety of scales added interest to the survey. Participants had a positive feeling towards numeric scales in general, and some had used them in previous surveys. A zero to ten scale was only used in the Brief Illness Perception Questionnaire [17] however it was the scale commented on the most by participants. Opinions varied from positive; "It's always really easy to understand that rating" (P11), to negative; it was "Difficult doing everything on a 1-10 scale of pain" (P12). The idea was raised that the scale had too many options and a smaller rating system would have been preferred. Some noted that they tended to put a neutral option on numeric scales and the option of 'not applicable' was also suggested. The reasoning behind the scales was considered by participants while completing the survey; "I wonder where this is coming from" (P10) and "Sometimes I thought I'd ticked things that contradicted each other" (P2). Specific scales were also discussed; the EQ-5D 0-100 visual analogue scale [18] asking people to rate their general health made participants reflect on their health. Response options such as "A good bit of the time, some of the time, a little of the time" in the MHI-5 measure of mental health status [19] were mentioned as being difficult to differentiate. Other specific scales are considered in the section on survey content.

### Theme 3: Survey content

#### Overall content

The overall impression was that the survey was very comprehensive with comments such as "No stone unturned" (P6) and completing the survey made participants reflect on their MSDs. However, there were additional aspects that participants wanted to include or expand. Specific suggestions were the opportunity to give background information about past MSDs, their work history and how their MSD happened, which treatments and treatment providers had been involved and to explain more about their MSD in general, for example if it was a short or long term injury. Others wanted the opportunity to explain about their work situation (for example if they were changing jobs), when they had sciatica or to explain that they had referred pain. Space for additional comments at the end of each anatomical site section was recommended. Methodological limitations of free-text comments in questionnaires have been discussed by Garcia et al (2004) however there are also ways in which information provided in this way might be useful, for example informing future research topics [20]. Parts of the survey were found to be repetitious but several people could see why this was and "I don't think you could change that at all" (P8). One participant suggested "List all of them [anatomical sites] at the top and go in the last twelve months did elbow you know, and dictate which ones you'd actually had time off work for" (P4). The survey seemed relevant and without an agenda which was important to participants; it was compared favourably to other surveys where questions appeared "Slanted" (P4) or "Stupid" (P11). Some questions did not apply to everyone and one participant did not see why the section on demographics was included although overall they thought the survey was relevant.

#### Time periods

The survey contained questions about the day the survey was completed, the previous seven days, one month and twelve months. This variety of timeframes meant participants had to carefully consider their answers but were better able to describe their MSD; "They were good because sometimes you might have said, you know, have you whatever in the last month, well no it's been fine in the last month but six months ago it was really bad" (P11). A twelve month recall period is commonly used in surveys of MSDs [21] and many participants had no problems recalling this timeframe; "Yeah pretty easy I think. Yeah, yeah I'd know if I was injured in the last year or the last week or the month" (P12). However, some commented on the difficulty of remembering back this far; "Well, it's really hard to remember back a year I guess" (P7), and, as mentioned previously, several participants had forgotten about the baseline survey when the follow-up survey arrived twelve months later. The time period participants believed recall would be reasonable ranged from three months to two years. Previous research has shown poor recall of sciatic pain after three years [22]. Participants felt it was easier to recall recent and more significant events, for example injuries and sudden onset MSDs (rather than those of gradual onset); "It was probably a bit harder to remember a year ago, or even when like a gradual injury, when that happened... [whereas]... when I had my, my accident I could pinpoint it to the day" (P14). It was also easier to recall information about the onset of MSDs if symptoms had been continuous rather than episodic.

#### Functional tasks

At each anatomical site participants were asked about their ability to get dressed and do the jobs they normally do around the house. Site specific questions were also asked, for example writing for wrist/hand pain and walking on level ground for knee pain. Three options were provided; 'no [difficulty]', 'difficult' or 'impossible.' Not everyone found this question clear to answer. Participants had problems answering these questions if they could undertake the task with pain or in a modified fashion, if it was only difficult occasionally or if only one household task was a problem; "Doing the jobs that you normally do around the house, well most of them are fine. There are the odd one or two that you find difficult... so you just put 'no' because there are that many tasks that you do around the house, that most of them are fine" (P1). In general, people felt the question adequately described the impact of their MSD; "No that sums it up. No, nothing I can think of there. That sums it up reasonably well I think" (P4). However, the addition of an open ended question was recommended. Other additions suggested were lifting a jug, gardening and vacuuming for arm pain, doing the laundry, showering or drying oneself for low back pain, a specific wrist movement involved in a work task, stability while lifting a weight (for example carrying things) for knee pain and leisure activities for MSDs in general.

#### Physical work tasks

Participants were asked whether they did eight physical work tasks on an average working day. There was an overall sense that these tasks reasonably described participants work; "I think that pretty much covers it" (P7). However there were some problems encountered, for example, difficulty interpreting thirty flights of stairs or feeling that four hours doing one task was too long; "Four hours to do one thing was quite a long time" (P14). Specific concepts mentioned were that someone might do a specific job for a long time on several occasions but not on "an average working day" and that the weight threshold of 25 kilograms was too high; people could be lifting a lower weight for a longer time; "I would have thought you should ask that at a lower weight... our trays are between eight and twelve kilo..it's quite intensive for short bursts, but you've actually shifted a fair bit of weight" (P4). One suggestion was to estimate the total weight lifted per day or the weight lifted and the duration. Specifying whether animate or inanimate objects were lifted was also recommended. Other ideas suggested for inclusion when assessing physical work tasks were aspects of the workstation, twisting, being on your feet, distances walked, driving and telephone work. Reaching was included in the questionnaire and the importance of this was acknowledged although the idea was raised that it could be clearer that this included reaching up and down as well as forwards.

#### Other specific aspects

It was mentioned that more than one box was applicable for the question regarding work schedule and, on reflection, the question does not state whether you can tick as many boxes are applicable or just one box that best describes your schedule.

Participants found it difficult to specify anatomical sites of MSDs if they had referred pain, for example if they had shoulder pain originating from their neck; "I think I answered more things about the neck, when I thought back I thought, oh no that's really down your shoulder" (P5). Another issue raised was that, although they may have had many minor MSDs, they only completed the survey for their more significant injuries.

In the survey, participants were asked whether they knew anyone else with MSDs. This raised the idea that, although friends or colleagues may have MSDs, they would not necessarily talk about them; "They're only relevant if people talk about it... 'cause a lot of people you know, you don't know" (P4).

### General Discussion

The three themes described in this study have the potential to contribute to knowledge on survey design. Randomised controlled trials have demonstrated that having an interesting topic increases response rate [7]. Participants in this qualitative study described reasons why they felt MSDs were interesting. For example, MSDs were applicable to themselves or others, they had observed unwanted consequences of MSDs so felt research was warranted, it aligned with their own natural or work interests and they felt the study had potential to benefit others. Although these specific ideas cannot be generalised to wider populations they give an insight into participants decision making. These ideas could be used when planning future studies as a basis for considering why a particular topic could interest potential participants and how best to convey these ideas.

Although the purpose of the paper was not to examine theories, aspects of the theme of participation align with the theory of social exchange [see [23]] where "...people are more likely to complete a mail questionnaire if they expect the costs to them of completing it are less than the expected rewards to themselves or groups with which they identify" (p. 176) [8]. In this study, some participants felt the survey was easy to complete, they had time to do it, they wanted to help others and they liked the monetary and non-monetary incentives. However, this research demonstrates a range of reasons why people took part, for example an enjoyment of surveys and satisfaction at feeling able to contribute in a meaningful way. When designing future surveys, acknowledging and addressing the variety of reasons why people might want to take part could enhance participation. Highlighting that people are invited to participate, whether or not they have the condition under investigation could reduce potential respondent bias (for example, if people would be more likely to participate if they had an MSD).

From the theme of survey design, participants' perspectives may contribute practically in the design of future surveys. For example, when providing information about a survey, the ability to miss non-applicable sections could be conveyed to potential participants at the outset as the lengthy appearance of a survey may be off-putting. The neutrality of a survey was also important and could be stressed in information accompanying a survey. Incentives were appreciated and compensated for less appealing features such as survey length.

Within the content of the survey itself, an explanation of why sections of information are being collected could be considered in future studies as knowing the relevance of specific questions was valued by participants. Specific areas suggested for expansion or addition, for example regarding physical and functional tasks, should be considered for future surveys on MSDs. If surveys consist of predominantly closed questions, a brief place for additional comments was recommended by participants in this study.

A potential limitation of the study was that only one of the fourteen participants was male. This does not represent a bias in responding to the invitation to participate in the qualitative interviews but reflects the underlying sampling frame. Of the twenty-two participants eligible for the qualitative study, the sole male invited did indeed participate. The underlying sampling frame was drawn from participants in the postal survey of which 86% were female at both baseline and follow-up. If males' views of survey design differ significantly from females this may not have been captured. Participants were drawn from specific geographical regions of New Zealand, Otago and Southland. Although there is no reason to believe responses on survey design would differ by location we cannot ascertain that from this study. Clearly, a further limitation is that, although this study provides information about participation in postal surveys, we do not know about those who did not take part. Given known difficulties approaching non-participants, we believe information about reasons for participation makes a useful contribution for future studies using postal surveys.

## Conclusion

This paper provides an insight into the perspectives of participants completing a postal survey and their reasons for participating in the study. Many concepts related to the need for clear and effective communication. These included the need for understandable and adequate information when inviting people to participate and clear communication within the survey itself. The ideas generated in this study may enhance the acceptability of future surveys to potential participants.

## Competing interests

The authors declare that they have no competing interests.

## Authors' contributions

HH planned the study, carried out the interviews, led the analysis undertaken for her PhD and led the preparation of the paper. SD participated in the design of the study, was present at the initial interview, helped plan the analysis, verified all coding and co-authored the paper. PH and DM participated in the design of the study and co-authored the paper. All authors read and approved the final manuscript.

## Authors' information

The material in this article is based on work towards HH's PhD Thesis. SD, PH and DM are Helen's PhD supervisors.

## Pre-publication history

The pre-publication history for this paper can be accessed here:

http://www.biomedcentral.com/1471-2288/11/8/prepub
